# Supplementary material for: Mechanical behavior of synthetic fiber ropes for mooring floating offshore wind turbines
Source: PLoS One. 2025 Feb 6;20(2):e0318190. doi: 10.1371/journal.pone.0318190 (PMC11801622; doi:10.1371/journal.pone.0318190)
Supplement: Attachment — Submitted filename: comments.doc [file pone.0318190.s002.doc]

Dear Editors and Reviewers:

Thank you for your letter and for the reviewers’ comments concerning our manuscript entitled “Study on the mechanical behaviour of synthetic fibre ropes in floating offshore wind turbine mooring systems”（PONE-D-24-36084）. Those comments are all valuable and very helpful for revising and improving our paper. We have studied reviewer’s comments carefully and have made revision which marked in red in the paper. The main corrections in the paper and the responds to the reviewer’s comments are as flowing:

**Reviewer #1:**

**1. The review’s comment:** It is hard to review since the figures are in the supplemental files not within the manuscript.

**The authors’ answer:**

Thank you for your valuable feedback. We are well aware of your concerns regarding placing data in the appendix rather than in the main text. This issue has been properly addressed, and we have integrated the collection of charts into the main text to present the information more clearly and facilitate the review process.

**2. The review’s comment:** Remove extra information from the abstract.

**The authors’ answer:**

Thank you for your suggestion. We have revised the abstract to make it more concise, clear, and to highlight the core content.

**3. The review’s comment:** Literature is not rich at all.

**The authors’ answer:**

Thank you for your suggestion. We have made modifications to the paper content and have found relevant institutions to assist with polishing. The relevant proof is as follows:

**4. The review’s comment:** I hardly see proper citations and references for the used equations.

**The authors’ answer:**

Thank you for your suggestion. We have recognized the deficiencies in the citation and references of the paper and have made corrections and additions to the formulas in the article.

**5. The review’s comment:** The results needs further discussion, do not only describe what you see.

**The authors’ answer:**

Thank you for your suggestion. We have re-described and supplemented the experimental results.Please refer to lines 240 to 249, 259 to 269, 269 to 289, 509 to 519 of the paper, and the conclusion section.

**6. The review’s comment:** The conclusion section should include contributions in past tense.

**The authors’ answer:**

Thank you for your suggestion. We have adjusted the tense of the conclusion section accordingly based on your proposal.

**7. The review’s comment:** Future recommendations should be addressed as well as limitations.

**The authors’ answer:**

Thank you for your suggestion. We have rephrased and modified the conclusion section.

**Reviewer #2:**

**1. The review’s comment:** Abstract: The abstract lacks the inclusion of key data and values from the experimental or analytical results. It is common practice to briefly summarize significant findings or numerical results in the abstract to provide a quick overview of the study's outcomes. The authors should include specific data points, performance metrics, or any significant findings from the tests conducted.

**The authors’ answer:**

Thank you for your feedback. We have rephrased the abstract of the paper, and the revised abstract includes a concise summary of the experimental content and key findings to provide a quick overview of the research results.

**2. The review’s comment:** Title and Material Selection: The title mentions "material selection," but the study does not appear to provide a comprehensive comparison or analysis of the materials in question (e.g., nylon and polyester ropes). While some discussion on the materials' performance exists, it is crucial to focus on the selection criteria and justification for choosing these materials as benchmarks.

**The authors’ answer:**

Thank you for your valuable feedback. We have recognized the inadequacies in the paper's title. This paper primarily focuses on experimental research on wear and stiffness of related mooring ropes, aiming to meet the requirements of mooring. Therefore, after considering your suggestion, we have adjusted the title of the paper accordingly to ensure its consistency with the research content.

**3. The review’s comment:** Literature Review: The number of reviewed papers is quite low. A more thorough examination of the existing literature is encouraged to establish the context for the research. Additionally, in many cases, references are grouped together, which can detract from a focused discussion of each source. It is better to reference individual papers, highlighting their relevance and contribution to the research.

**The authors’ answer:**

Thank you for your valuable feedback. We have rewritten the literature review section of the paper, reorganized the references, and increased the amount of literature read. We have cited more individual papers and emphasized the contributions of scholars' research.

**4. The review’s comment:** Coherence Between Title and Work: The paper lacks coherence between the title and the presented research. The focus on quasi-static stiffness tests is clear, but the connection to material selection, particularly in relation to Floating Wind Offshore Turbines (FWOTs), is not well established. The title should either be adjusted to reflect the primary focus of the work, or the research should be expanded to include a more comprehensive comparison of material selection for FWOTs.

**The authors’ answer:**

We sincerely thank you for your valuable suggestions. We have recognized the deficiencies in the paper's title and have made appropriate modifications. At the same time, we have re-articulated the conclusion section to ensure that the presentation of the research findings is clearer and more understandable. Please refer to the "Title" and "Conclusion" sections of the paper for details.

**5.The review’s comment:** Quasi-static Stiffness Tests: The authors present quasi-static stiffness tests on nylon and polyester ropes but fail to provide a comparative analysis or draw conclusions regarding material selection for FWOTs. It is strongly recommended that the authors conclude by analyzing previous data and justifying the selection of the superior material for FWOT applications.

**The authors’ answer:**

We sincerely thank you for your valuable suggestions. Your questions have played a significant role in improving our paper, and we have added the relevant conclusive content into the main text, which can be found on lines 509 to 519.

**6. The review’s comment:** Limitations in Measuring Yarn Angles (Lines 154-155): The authors briefly mention challenges in measuring angles between yarns, but the actual difficulties faced by the team are not discussed in detail. It is important to elaborate on the specific limitations encountered, whether they are technical, methodological, or equipment-related, to provide context for the challenges faced during the study.

**The authors’ answer:**

Thank you for your valuable feedback. We have thoroughly elaborated on the limitations encountered when measuring yarn angles, and the specific details can be found between lines 195 to 204 in the main text of the paper.

**7. The review’s comment:** Marine Oil Agents (Lines 216, 232): In multiple sections, the paper refers to "marine oil agents" without specifying which ones are being discussed. The authors should provide the necessary specifications, such as the chemical composition or brand of the agents, to improve clarity and reproducibility.

**The authors’ answer:**

Thank you for your valuable feedback. We have added the materials on the use of marine oil agents to the paper to ensure clarity and reproducibility of the experiments. Specific references can be found on lines 269 to 275.

**8. The review’s comment:** Equation 4: Equation 4 is left underdeveloped. The relations between the variables are not fully explained. It is important to clearly define the functional relationships between these variables and their influence on the quasi-static stiffness. Providing detailed explanations will enhance the readers' understanding of the mechanics behind the model.

**The authors’ answer:**

Thank you for your valuable feedback. We have provided a more detailed explanation of the derivation process, which can be found between lines 324 to 332.

**9. The review’s comment:** Equation 5: Equation 5 is incomplete as it lacks an equal sign. This needs to be corrected to properly represent it as an equation.

**The authors’ answer:**

We have revised equation 5 in the original paper; details can be found in equation 7.

**10. The review’s comment:** Best Fit Equations (Line 429): The authors mention the formation of "best fit equations" but do not elaborate on the methodology or criteria used for determining these equations. A brief explanation of how the equations were derived and why they are considered the best fit would provide greater transparency and credibility to the analysis.

**The authors’ answer:**

We sincerely thank you for your valuable feedback. During the data fitting process, we used MATLAB software to import data and utilized its automatic fitting feature. This allowed us to leverage MATLAB's powerful capabilities and advanced algorithms to analyze the data and determine the fitting curve or function that best reflects the characteristics of the data. This method helps us to more accurately grasp the relationships and trends within the data, thereby providing strong support for our decision-making and research in related fields.

**11. The review’s comment:** Figure Explanation (Fig 6(a), Fig 9, Fig 24): In Figure 6(a), the number 2418 is used in relation to twist, but this is not explained. The same number appears again in Figures 9 and 24. The authors should clarify the significance of 2418 and its connection to the data presented. Additionally, explain how this value relates to the observed twist behavior in the ropes.

**The authors’ answer:**

We sincerely appreciate the valuable feedback you have provided. Indeed, we have identified an issue in the PET fiber test condition 18: at the fixed knotting point, the yarn experienced breakage, resulting in a measured cycle count of only 2418. In response, we have updated the relevant details in the main text, which you can refer to on lines 217 to 223.

**12. The review’s comment:** Number of Figures: The number of figures in the paper is quite large. The authors should consider reducing the number of figures by combining related ones using multi-plot or another method. This would make the visual data more concise and improve the overall presentation without losing important insights.

**The authors’ answer:**

Thank you for your valuable feedback. We have made every effort to improve the icon issue by merging the original figures 9 and 10. To ensure the complete presentation of the experimental results, no adjustments have been made to the other charts.
