# Supplementary material for: Mechanical behavior of synthetic fiber ropes for mooring floating offshore wind turbines
Source: PLoS One. 2025 Feb 6;20(2):e0318190. doi: 10.1371/journal.pone.0318190 (PMC11801622; doi:10.1371/journal.pone.0318190)
Supplement: Attachment — Submitted filename: comments.docx [file pone.0318190.s003.docx]

Dear Editors and Reviewers:

Thank you for your letter and for the reviewers’ comments concerning our manuscript entitled “Mechanical behavior of synthetic fiber ropes for mooring floating offshore wind turbines”（PONE-D-24-36084R1）. Those comments are all valuable and very helpful for revising and improving our paper. We have studied reviewer’s comments carefully and have made revision which marked in red in the paper. The main corrections in the paper and the responds to the reviewer’s comments are as flowing:

**Additional Editor Comments:**

**1. The editor’s comment:** Define all the abbreviations on their first use.

**The authors’ answer:** Thank you for your suggestion. We have revised the acronyms; details can be found on line 107 of the article and in Table 1.

**2. The editor’s comment:** It is recommended to use high resolution images.

**The authors’ answer:** Thank you for your valuable feedback. The relevant figures have been updated. Please see the main text for reference.

**3. The editor’s comment:** What are the future recommendations for this research?

**The authors’ answer:** We sincerely appreciate the valuable feedback you have provided. We have supplemented our outlook for the future in lines 562 to 571 of the article. please refer to the main text for details.

**Reviewer #2:**

**1. The review’s comment:** The title has been improved, but it could be refined further for linguistic clarity and to make it more engaging and reader-friendly.

**The authors’ answer:** Thank you for your valuable suggestion. We have recognized the deficiencies in the paper's title and have made appropriate modifications to the title of the paper.

**2. The review’s comment:** While the results and discussions are much clearer now, a brief mention of the study's broader implications or real-world applications in the conclusion could enhance the manuscript's impact.

**The authors’ answer:** Thank you for your valuable feedback. We have added the corresponding content to lines 558-561 of the article. please refer to the main text for details.
